# Digital strategy and environmental performance: the mediating role of digitalization in SMEs

**DOI:** 10.1007/s44265-023-00010-5

**Published:** 2023-06-01

**Authors:** Inzamam Ul Haq, Chunhui Huo

**Affiliations:** Business School, Liaoning University, Shenyang, 110036 Liaoning Province China

**Keywords:** Digital strategy, Digitalization, Environmental performance, SMEs, Sustainability

## Abstract

**Purpose:**

Digitalization and digital strategy have become one of the variant concepts these days since the fourth industrial revolution 4.0. The earlier debate a on the role of digital strategy and degree of digitalization for environmental and sustainable performance of small and medium enterprises (SMEs) is at its nascent stages. This study aims to investigate the impact of digital strategy and digitalization on the environmental performance in Pakistani SME’s during the pandemic times. In addition, it explores the mediating role of five measures of digitalization degree such as overall digitalization degree (ODD), digitalization methods (OM), digital technology adaptation (DTA), digital product services (DPS) and digital processes (DP).

**Methods:**

This study collected data from 298 respondents using random sampling technique. The unit of analysis was managerial staff and employees working for the organization for last 5 years. To investigate the relationship between constructs, we considered Structural-equation modeling (SEM) using SMART-PLS.

**Results:**

Building on the contingency theory, the findings of the research reveal that digital strategy showed  a positive impact on digitalization measures but negatively impacted on overall environmental performance. In addition, (DP) ODD, OM, and DPS showed a (negative) positive meaningful impact on environmental performance and (partially) fully mediated the relationship between digital strategy and environmental performance. The digital strategy failed to improve the environmental performance however the role of digitalization is crucial to ensure environmental sustainability.

**Conclusions:**

Digital strategy alone fails to alleviate the pressure on environmental performance however excessive digitalization can aggravate the adverse consequences on environment. This research provides useful implications for governments and policymakers to avoid excessive digitalization.

**Supplementary Information:**

The online version contains supplementary material available at 10.1007/s44265-023-00010-5.

## Introduction

The importance of examining the digitalization of SMEs in Pakistan cannot be overstated. The State Bank of Pakistan highlighted several powerful reasons which are the key motivation of current research. First, the growth of the Small and Medium Enterprise sector is essential for the economy of the country. This sector of the economy is thought to be its backbone. Small and Medium Enterprise Development Authority (SMEDA) estimated that there are more than 5 million small and medium-sized enterprises (SMEs) in Pakistan. 25% of total exports and 40% of Pakistan's total export GDP are made up of SMEs. Second, after the agriculture sector, the SME sector employs the largest proportion of the working population in the country. The SME sector accounts for 78% of all employment in the non-agricultural sector. Finally, SMEs are a major force in the fight against poverty, national economic expansion, and job creation. Therefore, it has become one of the rapidly growing and functioning sections in Pakistan.

Focusing on previous research, SMEs are essential for fostering technical innovation, increasing employment opportunities, and upholding social stability (O'Regan et al. 2006). However, SMEs are significantly more susceptible to public or corporate crises and more difficult to remain sustainable and environment-friendly during these times (Mayr et al. 2017). In addition, due to the large number of SMEs, their role in environmental sustainability is crucial. In order to mitigate the threat of public crises on SMEs organizational and environmental performance, the research currently available has investigated the roles of production recovery, corporate social responsibility, dynamic capacities (organizational and employees and community involvement Ballesteros et al. 2017; Neise and Diez 2019; Proksch et al. 2021; Guo et al. 2020). Thus, it is surprising that there are very few empirical studies in related strands on the impact of a digital strategy on raising the level of digitalization in SMEs considering the sustainable concerns or environment sustainability in the present entrepreneurship literature (Proksch et al. 2021). This topic has received some attention in related areas in the field of small and medium-sized enterprises (Proksch et al. 2021; Guo et al. 2020; Mayr et al. 2017; Faridi and Malik 2020; Adomako et al. 2021; Lipsmeier et al. 2020; Isensee et al. 2020; Becker and Schmid 2020; Bi et al. 2019).

The debate on the sustainable concerns or environmental performance of SMEs considering the impact of digital strategy and digitalization is inclusive. Therefore, we provide a response to the research question of whether a digital strategy can raise the environmental performance and level of digitalization of SMEs by considering five perspectives of digitalization such as overall digitalization degree, digitalization methods, digital technology adaptation, digital, product service, and digital process combing both used by (Guo et al. 2020; Proksch et al. 2021). This may enable the organization to improve sustainable performance and perform better as a result. We used structural equation modelling (SEM) in the form of a partial least squares (PLS) technique to examine 384 respondents from SEMs sector of Pakistan.

Three key factors make it crucial to look into how a digital strategy affects the extent of SMEs digitalization degree. First, it responds to contemporary calls for research on the function of strategy and the application of digital technology in the context of entrepreneurship. Second, understanding how to use a digital strategy to produce more digital goods and services can assist SMEs in adding value for consumers while improving environmental performance. Third, knowing how a digital strategy affects the level of process digitalization helps SMEs identify opportunities for enhanced operational flexibility and efficiency, which can lead to cost advantages. Therefore, using a digital strategy to increase the degree of digitalization as well as environmental performance in SMEs may be a practical way for them to gain a competitive edge and, as a result, be able to attract customers through a sustainability focus (Shields and Shelleman 2015; El-Kassar and Singh 2019).

Our study contributes to the earlier literature in four major ways. First, it contributes to previous research (Guo et al. 2020; Proksch et al. 2021; Jin et al. 2021) in terms of considering five alternative measures to measure the degree of digitalization such as overall digitalization degree, digitalization methods, digital technology adaptation, digital product service, and digital process and their impact on environmental performance in sampled firms. Second, it adds that excessive digitalization is vulnerable to sustainable firm performance however previous research is more inclined to the positive outcome for companies, which could be positively biased (Proksch et al. 2021; Hadi and Baskaran 2021; Huong and Thanh 2022). Third, our study adds to earlier research (Guo et al. 2020; Proksch et al. 2021; Hervé et al. 2022; Xu et al. 2022; Shahzad et al. 2022) by studying the impact of digital strategy and degree of digitalization on sustainable firm performance of SMEs. Finally, the findings of current research add knowledge to the Contingency theory and answers to the important call of how digital strategy and environmental performance and strategy are aligned (Jin et al. 2021).

The remainder of the article is structured as follows. Section 2 discusses recent literature and the potential literature gap. Section 3 describes the survey and research methods. Section 4 compares and contrasts the findings of current research with discussed literature. Finally, the last section of the paper concludes the paper and suggests future direction for further research.

## Literature review

Nowadays, a digital strategy is almost as important as a corporate one. A digital strategy outlines a company’s overarching goals for digitization, together with the tactical steps needed to get there (Nayal et al. 2022). It outlines specific, short-medium and long-term digitization goals, and activities for a company’s structure and culture as well as for its goods, services and, value generation. A digital strategy is described as an organizational plan by (Bharadwaj et al. 2013) that makes use of digital resources to provide differentiating value. The digital strategy, in a limited sense, is built on automation and integration through cutting-edge software and hardware solutions, as well as through the improvement of computer networks and the implementation of relevant protocols, standards, and rules (Ivanova 2020). According to (Chi et al. 2016), a digital business strategy is a type of firm-level strategy that affects how digital resources, and capabilities are used. We define the construct of digital strategy as per the definition of Chi et al. (2016).

“Digitalization” is a variant term (Huong and Thanh 2022) that refers to a shift that is more fundamental than merely digitizing current workflows or outputs. Digitalization is actually “the acceptance or increase in usage of digital or computer technology by an organization, industry, and country” (Brennen and Kreiss 2014). Similar to this, it was documented that (Bleicher and Stanley 2016) that digitalization is the process of covering data and information from analogue to digital format. Even though the significance of digitalization has been acknowledged and established however small and medium industries (SMEs) sometimes struggle to comprehend its potential effects and advantages (Trabert et al. 2022).

Digitalization is predicted to have significant global economic, financial, and social consequences. This includes the creation, use, and disposal of digital technologies and applications, including robotics, the Internet of Things (IoT), distributed ledger technologies like blockchain, and artificial intelligence, as well as hardware (such as information and communication technologies (ICT) equipment, data centers, and data transmission networks). Digitalization is causing changes in the employment, workplace, connections, and skills required working in SMEs. However, businesses want to make more money, and it has been seen that the more digitalization a business adopts, the higher its level of digitalization and the greater its emphasis on digital goods, services and procedures (Proksch et al. 2021).

Even though modern technological approaches require significant expenditures, by embracing new possibilities and altering their business practices, SMEs may use these methods to gain a competitive edge in a highly competitive global market (Adomako et al. 2021; El-Kassar and Singh 2019). Although using digital technology in SMEs presents significant challenges, it also offers a number of opportunities. According to Faridi and Malik (2020), larger organizations have the potential and capability to become digital innovators compared to small industries with lower capital and size. because the adoption of digital technology depends on the firm’s size and culture. Additionally, sensors and transmission equipment decreasing prices and wide market penetration have sped up the digitalization process. In this approach, cost-effectiveness and market enlargement are made possible by digital product-service platforms (Simonsson et al. 2020). In general, it has been well-developed that advancements in digital technology have increased the effectiveness of businesses’ services and goods (Nylén and Holmström, 2015; Reim et al. 2015; de la Calle et al. 2021). Due to their significant impact on countries and regions, SMEs should transform their organizational structures and business-making cultures, starting with manufacturing technologies and management paradigms, in order to achieve a successful digital transformation process. This is because digital processes include encourages those activities that add value (Awinja and Fatoki 2021).

Although, a company’s ability to achieve its aims and objectives is measured by its performance when compared to its main rivals (Cao and Zhang 2011). In addition, profitability, growth, and market value are often characteristics of outstanding corporate performance (Cho and Pucik 2005). Over the past few years, digitization has drawn more and more attention (Calvard and Jeske 2018). The performance of a business, however, has been shown to be significantly influenced by digitalization, and higher levels of digitalization have resulted in improved firm performance (Nwankpa and Roumani 2016). However, how digitalization process and digital strategy influence sustainable practices throughout the organization or SMEs. Environmental performance is key to businesses to attract new customers and stakeholders. More importantly, environmental performance has become a serious concern to protect, sustain and preserve the environment. Environmental performance is a diverse construct and scholars have defined it differently in diverse contexts (El-Kassar and Singh 2019). This study considers the concept of environmental performance as used by previous research by El-Kassar and Singh (2019). They defined environmental performance focusing on the definition of Lin et al. (2013). Lin et al. (2013) defined environmental performance the reduction in hazardous waste and emissions, partnering with green suppliers, use of environment-friendly materials, and compliance with environmental criteria. Business practices around the globe is worsening environmental quality and increasing carbon footprints and global warming (Ul Haq et al.  2022a, b ). Therefore, it is more important to study the impact of digital strategy and digitalization on environmental performance through considering the sustainable and environment-friendly practices.

### Digital strategy and environmental performance

Firms experience unique modes of value generation and appropriation as a result of digital business strategy. Despite digitalization prospects in SMEs, few enterprises have digitalized their production or established new revenue models (Chi et al. 2018). Significant modifications have been made to the products, procedures, structures, and systems. According to the literature, the goal of the digital transformation strategy is to create distinctive value using digital resources (Bharadwaj et al. 2013). Despite having made identical investments in digitization, various firms in different areas show distinct payoffs (Dhar and Sundararajan 2007). According to Martínez-Caro et al. (2020) creating a digital strategy makes it easier for businesses to digitize themselves and to create value from their use of digital technologies, which will ultimately lead to better organizational performance. A digital strategy is an important tool utilized by top management, and in the case of SMEs, digital strategy improves SMEs' performance by leveraging existing resources and promotional efforts (Tan et al. 2015). According to Joensuu-Salo et al. (2017), digitalization has a considerable impact on company performance, particularly that connected to business development. Previous research has indicated that effective digital strategy and digital transformation initiatives resulted in improved and long-term corporate performance (Dalenogare et al. 2018, Vial 2021). This demonstrates that organizations that use more digitally integrated business processes benefit from improved performance and competitive a edge in a market (El-Kassar and Singh 2019).

Similar to the impact of digital strategy concerns on traditional overall business performance, the digital strategy showed favorable consequences for environmental performance. For instance, Huong and Thanh (2022) empirically examined the effects of digitalization on the environmental performance index and environmental health index in 25 European countries from 2015 through 2020 using secondary data. They documented a positive relationship between digitalization and both environmental indexes such as environmental performance index and environmental health index. Although this relationship appears to be indirect and ambiguous, digital technologies are helping to reduce pressure on the natural environment and biodiversity, which are indicators of ecosystem vitality (Liu et al. 2019; Huong and Thanh 2022). Notably, El-Kassar and Singh (2019) argued that only green innovation and green technologies foster the firm green performance as well as a competitive advantage of companies. However, it remains an important question that how digital strategy and environmental performance are aligned. In order to study the relationship between digital strategy and environmental performance we proposed the following hypothesis,

H1: The digital strategy has a significant impact on the environmental performance.

### Digital strategy and digitalization

A digital strategy is one of the most important tools for incorporating digitalization in businesses for those that want to be successful in the digital era (Kim et al. 2013). In order to attain the intended future state of being digitally changed, a predigital organization’s transformation initiatives are expected to be coordinated, prioritized, and implemented (Matt et al. 2015). Consequently, a digital transformation plan aims to offer insights into how such a company-wide digital strategy may be created and implemented (Hess et al. 2016). The extent to which changes brought about by digital technology have affected an organization is reflected in its digital strategy. The majority of thinking in the field of digitalization is likewise driven by the notion of using digital technology to better business outcomes (Vial 2021). Digitalization presents SMEs with prospects for expansion and globalization (Joensuu-Salo et al. 2017). A digital strategy developed by management assists in coordinating a number of organizational aspects to raise the level of digitalization in terms of digital processes and products (Symeonidou and Nicolaou 2018). von Briel et al. (2018) argued that by enhancing organizational flexibility and resilience, digitalization aids businesses in gaining and maintaining competitive advantages. The existing literature has previously emphasized digitization as a significant component of businesses’ strategic direction (Lipsmeier et al. 2020; Proksch et al. 2021; Becker and Schmid 2020; Eller et al. 2020). Kim et al. (2013) even emphasized that a digital strategy is one of the most important factors in adopting digitalization in organizations. Importantly, having only a digital strategy in place is unable to achieve a higher degree of digitalization (Proksch et al. 2021) considering only two measures of digitalization. Therefore, previous research provides contradictory findings on the link between digital strategy and digitalization either direct or indirect effects of digital strategy on degree of digitalization. Focusing on the five measures of digitalization, we proposed the following hypothesis,

H2: The digital strategy has a significant impact on each measure of digitalization i.e., ODD (H2a), DM (H2b), DTA (H2c), DPS (H2d) and DP (H2e).

### Digital strategy, digitalization and environmental performance

Previous research has established a strong connection between digital strategy and the degree of digitization (Szedlak et al. 2022; Högberg and Willermark 2022). In this research, the contingency theory enables to look at the link between digital strategy and digitalization considering the five areas i.e., overall digitalization degree, digitalization methods, digital technology adaptation (Guo et al. 2020), digital products or services and digital processes (Proksch et al. 2021) among SMEs in Pakistan. Digitalization involves the usage of digital technologies, in product manufacturing, services, and process to optimize the benefits (Proksch et al. 2021). Previous research has highlighted two core reasons for a strong relationship between digital strategy and the digitalization process. First, the potential opportunities of digital strategies to innovate more digital products and services which add value to the customer as well as a product (Rachinger et al. 2018). Second, the role of digital strategy toward the degree of digitalization optimizes the processes and fosters operational flexibility and efficiency (Nambisan 2017). Kim et al. (2013) argued that digital strategy is one of the key sources of incorporating and promoting digitalization in businesses. In contrast, achieving a higher degree of digitalization is insufficient through digital strategy only (Proksch et al. 2021). In addition, it preserves the environment and resources (tangible and intelligible) (Moreno‐Moya and Munuera‐Aleman 2016, Isensee et al. 2020; Eller et al. 2020). More recently, digitalization is a major driver to improved the environmental performance particularly in European countries (Huong and Thanh 2022). More specifically, Jin et al. (2021) investigated the impact of information communication technology (ICT) on environmental performance and they found that ICT capabilities and environmental performance have a meaningful positive relationship in Chinese settings. Similarly, digital culture also enhances sustainable practices and performance (Hadi and Baskaran 2021). Overall, digital technologies may in a variety of ways serve to reduce pressure on the environment and enhance biodiversity (Huong and Thanh 2022). In overview, previous research has been focusing on the impact of digital strategy and digitalization on the overall business performance i.e., financial performance and in terms of competitive edge (El-Kassar and Singh 2019). Meanwhile, previous research found that digitalization of business processes may harm the environment or sustainability due to later inception and introduction, however, the digitalization has ample potential to sustain and protect the environment through socially responsible practices (Ahmadova et al. 2022). However rare research investigated the relationship between digital strategy, digitalization and environmental performance in current settings. In order to do so, we propose,

H3: Each measure of digitalization i.e., ODD (H3a), DM (H3b), DTA (H3c), DPS (H3d), and DP (H3e) has significant impact on the environmental performance.

H4: Each measure of digitalization i.e., ODD (H4a), DM (H4b), DTA (H4c), DPS (H4d), and DP (H4e) mediates the link between digital strategy and environmental performance.

**Fig. 1:**
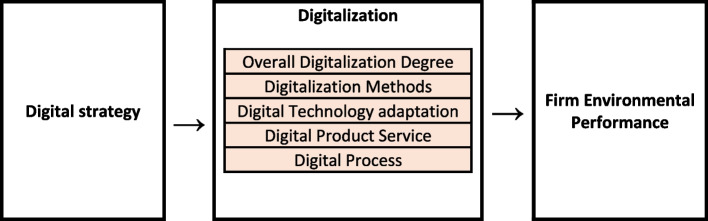
Theoretical framework

On the basis of literature gap and current literature discussion we proposed our theoretical framework, Fig. 1 summarizes the theoretical framework of the study.

## Survey and research methods

### Sample and data collection

Our study focuses on smalland medium enterprises (SMEs) in Pakistan. In this article, we define SMEs on the basis of number of employees. The small-sized firm has up-to 50 workers and medium-sized enterprises have more than 50 but up-to 250 workers. Studying the digitalization of SMEs in Pakistan is crucial for a number of reasons. First, the Small and Medium Enterprise sector is crucial to the nation's economic growth. This industry is regarded as the foundation of any economy. More than 5 million small and medium-sized businesses (SMEs) are estimated to exist in Pakistan, according to SMEDA. SMEs make up 25% of all exports and 40% of Pakistan's total export GDP. Second, The SME sector employs the biggest percentage of the working people in the nation, second only to the agriculture industry. Overall, 78% of employment in the non-agricultural sector comes from the SME sector. Finally, one of the key drivers of poverty reduction, national economic growth, and job creation is SMEs. Table 2 demonstrates the results of descriptive statistics. Our study tested mean, standard deviation, skewness, kurtosis, minimum, and maximum as descriptive statistics. The results of descriptive statistics are satisfactory and express the nature of the data.

The data was collected through a random sampling technique through the surveys. In short, the questionnaire was designed using Google Forms online and the online questionnaire was used and shared with SMEs' managers/owners via WhatsApp and Gmail to collect first-hand data during the pandemic time (March 2022 to July 2022). In total 437 responses were collected and 384 were considered for analysis, removed rest of the responses were due to issues i.e., incomplete responses and wrongly filled, etc. to ensure reliable PLS-SEM analysis.

In order to create a model with a high degree of reliability, following El-Kassar and Singh (2019) we used a method that was adapted from Davidsson et al. (2017). We first improved the items reflected and adapted from the literature. Furthermore, two experts from the digitalization field were asked to highlight potential areas for improvement to minimize the bias while data collection. After correcting highlighted issues, the questionnaire was refined and finalized for data collection, and we made changes to the survey questions in response to their input.

Table 1 reported the respondents' demographic information. Generally, responders were dominated by masculine-managerial positions where 77.60 percent were male, and 22.40 percent were female. Being youth-dominated business ventures more than 80 percent were between the age of 21 to 35 years. Interestingly, respondents are well-educated however 38.54 percent had only bachelor’s degrees in diverse disciplines. Major respondents belong to industrial and well-developed cities of Pakistan i.e., twin cities Islamabad-Rawalpindi, Karachi, Lahore, and Sialkot where around half of the respondents were based in Islamabad-Rawalpindi.

**Table 1:** Summary of demographics

Details	Respondents	%
**Gender**		
*Male*	298	77.60%
*Female*	86	22.40%
**Age**		
*21 to 25 years*	43	11.20%
*26 to 30 years*	142	36.98%
*31 to 35 years*	132	34.38%
*36 to 40 years*	41	10.68%
*41 to 45 years*	26	6.77%
**Education**		
*MBA*	112	29.17%
*EMBA*	84	21.88%
*Bachelor*	148	38.54%
*Diploma and others*	40	10.42%
**City**		
*Islamabad-Rawalpindi*	189	49.22%
*Karachi*	124	32.29%
*Lahore*	100	26.04%
*Sialkot*	71	18.49%
**Total**	*n* = 484	100%

### Measures

All constructs in the theoretical model were assessed using the items. Five-point Likert scale was used for all variables. Table 2 reported items for constructs. This study used three core constructs such as digital strategy, digitalization, and environmental performance. Digital strategy is an exogenous construct which is reflected through five items scale used by Proksch et al. (2021) and initially adapted from (Kim et al. 2013; Hakala and Kohtamäki 2011; Chen et al. 2014). Five alternative measures were utilized to measure the degree of digitalization of SMEs in Pakistan. Overall digitalization degree, business mode or method, and adaptation of digital technologies where measures were adapted from the relevant study from the digitization domain (Guo et al. 2020) where overall digitalization degree, digitalization methods, digital technology adaptation have five-items, two-items and seven-items scales. The rest of the two measures of digitization i.e., digital product services and digital processes were adapted from Proksch et al. (2021) having six-items for each measure. Finally, the measure of environmental performance was sourced from El-Kassar and Singh (2019) and reflected through six items scale. All these measures were considered from studies which are concerning digitization and digital strategy.

**Table 2:** Factor loadings

Item	Digital Strategy	Overall Digitalization Degree	Digitalization Methods	Digital Technology adaptation	Digital Product Service	Digital Process	Environmental Performance
DS1	0.788						
DS2	0.717						
DS3	0.717						
DS4	0.960						
DS5	0.828						
ODD1		0.848					
ODD2		0.818					
ODD3		0.960					
ODD4		0.869					
ODD5		0.879					
DM1			0.727				
DM2			0.798				
DTA1				0.879			
DTA2				0.758			
DTA3				0.808			
DTA4				0.848			
DTA5				0.848			
DTA6				0.889			
DTA7				0.808			
DPS1					0.707		
DPS2					0.747		
DPS3					0.859		
DPS4					0.778		
DPS5					0.919		
DP1						0.929	
DP2						0.889	
DP3						0.828	
DP4						0.960	
DP5						0.899	
EP1							0.929
EP2							0.778
EP3							0.737
EP4							0.960
EP5							0.919
EP6							0.838

Our research provides both data collection and statistical tests in the analysis section to mitigate the common bias risk. Firstly, we informed the respondents that their replies would be kept private and that there were no correct or incorrect answers. Secondly, we relied on established measures, which may also diminish the common bias method (Podsakoff et al. 2003). Finally, we applied the variance inflation factor (VIF) as a measure to test the common bias. The range of VIF coefficients was from 1.078 to 3.018, indicating the absence of common method bias as the threshold of VIF is 5 (Kock 2015).

## Analysis and results

### Statistical analysis

We followed Structural Equation Modeling (SEM) analysis to test the proposed direct and indirect effects or paths in the model. Our study focused on the Partial least square (PLS) form of Structural Equation Modeling which has enormous advantages over other methods (Hair Jr et al. 2017). In addition, PLS-SEM has gained popularity in recent literature (Haq and Awan 2020; Hair et al. 2011). The PLS-SEM model is based on two models (El-Kassar and Singh 2019). The constructs and their items are included in the outer model, while the relations between the constructs are included in the inner model. Following previous studies (El-Kassar and Singh 2019, Ul Haq et al. 2022a) we estimated the model's parameters in three steps. Initial iterations of the algorithm's score estimation for the latent variable (the score for each construct i.e., Cronbach alpha, construct reliability, Average-Variance Extracted, Heterotrait-Monotrait ratio). Second, the algorithm calculates the outer weights/loadings. Third, regarding the path direct and indirect coefficients, we estimated path analysis using Bootstrap (using multivariate linear regression).^1^

There are several advantages underpining the PLS-SEM analysis technique. First, PLS-SEM is a powerful technique for exploratory study targeted at developing new theories or outcomes (Haq and Awan 2020). Because models that assess the level of digitization of new businesses are currently lacking in entrepreneurship research, our study can be regarded as exploratory. Second, PLS-SEM models are preferred when the study objective is to explain the model's variation (Hair Jr et al. 2017). Third, PLS-SEM can produce feasible output even with a small sample size (Huang et al. 2015). We attempted to optimize the explained variation in our study of the impact of a digital strategy as well as other elements that might have an impact on the level of digitalization and environmental performance. The estimation process of the PLS-SEM model was performed in Smart-PLS software (version 3.3.2) using the default properties. We used a reflective measurement mode for all our constructs. As the hypothesis of the study is to investigate the positive or negative but significant relationship between constructs, therefore, a two-tailed test type was used.

### Results

#### Measurement model results

Based on 5,000 bootstrapping, Table 2 displays the factors loadings for all indicators of each construct. Factor loading is a measure of indicator or item reliability in the model. All values of factor loadings are above the threshold point of 0.70 (Hair Jr et al. 2021). This suggests that all items are reliable hence indicator reliability is maintained. Overall, all items are significant at a 1 percent (1%) significance level, hence indicatory reliability is highly satisfactory. Additionally, we used Cronbach's alpha, composite reliability, and the AVE to evaluate the reliability of our constructs as the findings are reported in Table 3. All of our constructs exceeded the Cronbach's alpha cutoff of 0.70 that we employed (Hair Jr et al. 2021). Additionally, all constructions exceeded the AVE criteria of 0.50 as well as the composite reliability requirement of 0.70 (Hair Jr et al. 2021). We come to the conclusion that our constructs are reliable and valid measures.

**Table 3:** Descriptive and Reliability analysis

Constructs	Alpha	CR	AVE	M	SD	Skew	Kurt	Min	Max	VIF
DS	0.910	0.933	0.742	4.534	0.763	-2.733	10.306	1	5	2.441
ODD	0.818	0.900	0.726	4.495	0.810	-2.656	9.161	1	5	1.212
DM	0.895	0.921	0.841	4.435	0.900	-2.522	7.264	1	5	2.441
DTA	0.862	0.832	0.811	4.499	0.769	-2.519	9.081	1	5	2.571
DPS	0.834	0.706	0.762	4.478	0.810	-2.538	8.602	1	5	3.018
DP	0.892	0.966	0.859	4.444	0.871	-2.534	7.725	1	5	2.401
EP	0.841	0.993	0.557	4.445	0.874	-2.551	7.698	1	5	1.078

*DS* Digital Strategy, *ODD* Overall Digitalization Degree, *DM* Digitalization Methods, *DTA* Digital Technology Adaptation, *DPS* Digital Product Service, *DP* Digital Process, *EP* Environmental Performance, *CR* Construct reliability, *AVE* Average Variance Extracted, *M* Mean, *SD* Standard Deviation, *Skew* Skewness, *Kurt* Kurtosis, *Min* Minimum, *Max* Maximum

The results of Table 4 indicated the correlation analysis or HTMT ratio. Coefficients should be lower than 0.80 according to the threshold (Hair Jr et al. 2021). Each coefficient indicates the correlation of one variable with the other. A higher HTMT ratio or correlation than 0.80 indicates the issue of high similarity between constract measures. As, all correlation coefficient is lower than 0.80 hence the discriminant validity is maintained.

**Table 4:** Heterotrait-Monotrait ratio

	**DS**	**ODD**	**DM**	**DTA**	**DPS**	**DP**	**EP**
DS	**-**						
ODD	0.518	**-**					
DM	0.495	0.411	**-**				
DTA	0.362	0.620	0.455	**-**			
DPS	0.434	0.346	0.412	0.552	**-**		
DP	0.592	0.452	0.152	0.347	0.445	**-**	
EP	0.141	0.322	0.124	0.543	0.118	0.231	**-**

#### Structural model results

**Fig. 2:**
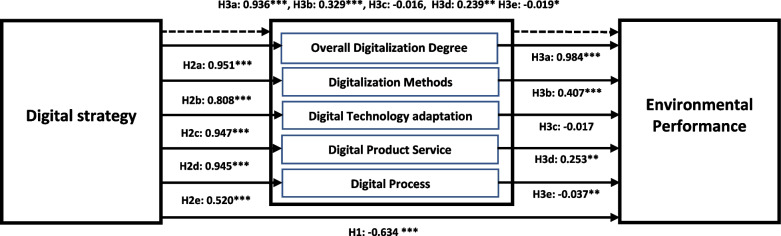
Structural Equation Model Output

In order to test the hypothesis, we estimated path analysis based on direct and indirect effects. In total, there were 4 hypotheses were proposed, 3 were direct effects and 1 was indirect or mediating hypothesis. The results for the direct and indirect effects are reported in Table 5 and Fig. 2. Focusing on the direct effects or path coefficient, $\beta = -0.634\ and\ p-value = 0.000$ showed that digital strategy has a significant but negative effect on environmental performance. Hence hypothesis H1 was accepted. Furthermore, the second hypothesis is based on five sub-hypotheses. Five sub-hypotheses tested the impact of digital strategy on five measures of digitization individually. Overall, digital strategy has a positive significant impact on overall digitalization degree, digitalization methods, digital, technology adaptation, digital product service, and digital process where $\beta = 0.951\ and\ p-value = 0.000$, $\beta = 0.808\ and\ p-value = 0.000$, $\beta = 0.947\ and\ p-value = 0.000$, $\beta = 0.945\ and\ p-value = 0.000$ and $\beta = 0.520\ and\ p-value = 0.000$. Hence, hypothesis 2 (H2a, H2b, H2c, H2d, and H2e) were accepted. Notably, digital strategy has the strongest (weakest) impact on digital methods (digital process). Focusing on the direct effect of five digitalization measures, overall digitalization degree, digitalization methods, and digital product service have a positive significant impact on environmental performance $\beta = 0.984\ and\ p-value = 0.000$, $\beta = 0.407\ and\ p-value = 0.000$, $\beta = 0.253 and p-value = 0.000$. Hence, hypotheses 3 (H3a, H3b, and H3d) were accepted partially. On the other hand, digital technology adaptation showed an insignificant impact on environmental performance $\beta = -0.017\ and\ p-value = 0.444$ and digital process showed a negative significant impact on environmental performance $\beta = -0.037\ and\ p-value = 0.043$. Therefore, subsequent results rejected the hypothesis H3c and accepted H3e .

**Table 5:** Direct and Indirect effects

Hypothesis	Path	O	M	SD	T Statistic	P Values	Result
H1	DS → EP	-0.634	-0.618	0.162	3.133	0.000	Accepted
H2a	DS → ODD	0.951	0.959	0.011	88.006	0.000	Accepted
H2b	DS → DM	0.808	0.835	0.048	17.884	0.000	Accepted
H2c	DS → DTA	0.947	0.945	0.016	59.296	0.000	Accepted
H2d	DS → DPS	0.945	0.950	0.014	69.668	0.000	Accepted
H2e	DS → DP	0.520	0.557	0.115	4.424	0.000	Accepted
H3a	ODD → EP	0.984	1.003	0.205	3.003	0.000	Accepted
H3b	DM → EP	0.407	0.421	0.118	3.355	0.000	Accepted
H3c	DTA → EP	-0.017	-0.052	0.117	0.089	0.444	Rejected
H3d	DPS → EP	0.253	0.241	0.133	1.783	0.029	Accepted
H3e	DP → EP	-0.037	-0.032	0.022	1.164	0.043	Accepted
H4a	DS → ODD → EP	0.936	0.962	0.198	4.731	0.000	Accepted
H4b	DS → DM → EP	0.329	0.351	0.100	3.298	0.001	Accepted
H4c	DS → DTA → EP	-0.016	-0.049	0.112	0.141	0.444	Rejected
H4d	DS → DPS → EP	0.239	0.230	0.128	1.869	0.031	Accepted
H4e	DS → DP → EP	-0.019	-0.018	0.014	1.421	0.078	Accepted

*Note: Original sample or *$\beta$* (O), Sample Mean (M) and Standard deviation (SD)*

Focusing on the indirect effects or mediating role of digitalization measures, we tested the mediating role of five digitalization measures between digital strategy, and environmental performance. Overall digitalization degree, digitalization methods and digital product service have significant positive mediating role between digital strategy and environmental performance. For instance, $\beta = 0.936\ and\ p-value = 0.000$, $\beta = 0.329\ and\ p-value = 0.000$  *and* $\beta = 0.239\ and\ p-value = 0.000$ confirmed that sub-hypotheses H4a, H4b and H4d were accepted hence, it proved full mediation. Contrary to this, digital technology adaptation failed to meditate the link between digital strategy and environmental performance where $\beta = -0.016\ and\ p-value = 0.444$ and digital process partially mediated the linkage as $\beta = -0.019\ and\ p-value = 0.078$ showed significant indirect negative effect, hence mediation sub-hypothesis H4c was rejected and H4e was accepted.

## Discussion

Our research findings are supportive of our theoretical model in order to understand the role of digital strategy to foster the digitalization degree and environmental performance of SMEs in Pakistan. We studied the degree of digitalization using five diverse perspectives such as overall digitalization degree, digitalization methods, digital technology adaptation, digital product service, and digital process. In particular, this paper studied the direct impact of digital strategy on digitalization and environmental performance. We looked into the mediating role of digitalization measures of overall digitalization degree, digitalization methods, digital technology adaptation, digital product service, and digital process between digital strategy and environmental performance which contributes to the earlier research (Proksch et al. 2021; Guo et al. 2020).

One of the major findings and contributions of our research is that digital strategy negatively predicts environmental performance. In other words, digital strategy alone fails to alleviate the pressure on environmental performance but aggravates the adverse consequences on the environmental performance. This finding can also be explained by the fact that the strategic introduction of digital technologies is increasing pressure on the natural environment (Liu et al. 2019). The adaptation of digital technologies and applications such as robotics, IoT, blockchain, and AI and ICT has increased the vulnerability to environmental and sustainability issues (Berkhout and Hertin 2004; Isensee et al. 2020). Our research reveals a positive direct relationship between the digital strategy and digitalization measures which corroborates with Proksch et al. (2021) who documented that the role of digitalization is crucial to optimize the influence of digital strategy. Additionally, the increased use of digital technologies creates new economic prospects that enable businesses to optimize their digitalization operations (Sebastian et al. 2020). There might be two core reasons behind the positive linkage between these constructs. For instance, using the digital strategy to produce more digital goods and services can assist SMEs in adding value for consumers (Rachinger et al. 2018). Second, digital strategy affects the level of process digitalization and helps SMEs identify opportunities for enhanced operational flexibility and efficiency, which can lead to cost-advantages (Nambisan 2017).

The role of digitalization measures (ODD, DS, and DPS) toward environmental performance is favorable which contributes to the earlier research which finds that digitalization is a major driver to improve the environmental performance particularly in European countries (Huong and Thanh 2022). These findings also support the idea of Huong and Thanh (2022) that digital technologies may in a variety of ways serve to reduce pressure on the environment and enhance biodiversity. Therefore, mare digital strategy might fail to optimize environmental performance but requires practical implementation of digitalization strategy into practice. Moreover, digitalization fosters positive environmental performance but it is uncertain and indirect (Huong and Thanh 2022). Similarly, digital culture also enhances sustainable practices and performance (Hadi and Baskaran 2021). Contrary to this discussion, digital processes (a measure of digitalization) might improve the overall productivity of the firm and competitive edge (Tsou and Hsu 2015; El-Kassar and Singh 2019) however digital processes minimizes the environmental performance due to pollution and high energy consumption. Considering the role of digital strategy and digitalization perspectives on environmental performance. Our findings are contradicting earlier research (Proksch et al. 2021; Hadi and Baskaran 2021; Huong and Thanh 2022) which documented that digital strategy may have a positive influence on environmental performance. To obtain a favorable outcome in terms of environmental sustainability, both digital strategy and digitalization are indispensable. In addition, strategic decisions regarding digitalization are causing excessive digitalization which fosters pollution and carbon emissions and damages the environmental performance (Ahmadova et al. 2022).

## Conclusion and future direction

The COVID-19 epidemic is a public health emergency that has made it extremely difficult for SMEs to survive and develop in current times. The situation has also brought to light the crucial part played by digital technology toward sustainability or environmental performance during COVID-19. This study examined the direct linkages between digital strategy and environmental performance; mediating roles of five digitalization measures of SMEs in Pakistan using data from a questionnaire through a survey technique.

This major contribution of the study is the negative impact of digital strategy on SME’s environmental performance directly. This finding implies that excessive digital strategies and strategic decisions of SMEs might harm the environment and sustainable development. This finding also reveals that a digital strategy fosters digitalization efforts, as demonstrated by their five measures: degree of digitalization, use of digital technology, business model, digital product service, and digital process. Further, our study looked at five mediators in the relationship between digital strategy and environmental performance: degree of digitalization, use of digital technology, business model, digital product service, and digital process. Importantly, the adverse impact of excessive digital strategy and decisions on environmental performance can be fully mediated by digitalization, digitalization methods, digital product, or service. It suggests that the role of digitalization is crucial to successfully preserve and protect environmental performance from digital strategic decisions which can aid in responding more effectively to sustainable concerns.

Current research is not without limitations. For instance, this research is carried out in Pakistan and data was collected from the SMEs sector. Hence, the findings of the present research cannot be generalized for developing economies or sectors i.e., information technology and e-commerce, etc. This study is cross-sectional research where data was collected during the global health crisis due to COVID-19. Finally, the data of current research takes data into account from different respondents who share demographical backgrounds. However, current research does not focus on testing group differences. For future research, more interesting results may be produced by testing group differences concerning the main hypotheses. Moreover, scholars and students need to retest this model in other national settings i.e., China, Bangladesh, and India. In addition, potential moderating variables might be tested in the current model, such as digital culture to further refine or uncover the role of enterprise aspects toward environmental performance.

## Supplementary Information

**Additional file 1. **Questionnaire.

## Footnotes

1 See Hair et al. (2011); Haq and Awan (2020) for more comprehensive information on PLS and its application.
