# Supplementary material for: Digital strategy and environmental performance: the mediating role of digitalization in SMEs
Source: DESD. 2023 Jun 1;1(1):9. doi: 10.1007/s44265-023-00010-5 (PMC10232346; doi:10.1007/s44265-023-00010-5)
Supplement: Supplementary file 1 — Additional file 1. Questionnaire. [file 44265_2023_10_MOESM1_ESM.docx]

**Appendix**

**Questionnaire**

I am student of Ph.D. Enterprise Management student in business school, Liaoning University, China. The survey is designed to collect primary data of Pakistani Small Medium Enterprises from managerial staff and employees working. I need your observations and true opinion by filling this questionnaire. There is no moralities or incorrect answers to questions offered. I am only fascinated to your true and genuine point of view. It is surety that the information given by you will be kept confidential and will be only used for academic purpose.

**Demographic Details**

| **GENDER** | | | | | ▣ Male | | | | ▣ Female | |
| --- | --- | --- | --- | --- | --- | --- | --- | --- | --- | --- |
| **AGE** | ▣ 21 to 25 years | ▣ 21 to 25 years | ▣ 21 to 25 years | | | | ▣ 21 to 25 years | | | ▣ 21 to 25 years |
| **EDUCATION** | ▣ MBA | ▣ EMBA | | ▣ Bachelor | | | | ▣ Diploma or Others | | |
| **CITY** | ▣ Islamabad-Rawalpindi | ▣ Karachi | ▣ Lahore | | | | | ▣ Sialkot | | |
| **Do you have minimum of five years of experience in the organization?** | | | | | | ▣ Yes | | | ▣ No | |

Please read each question carefully and tick the most suitable answer in the box below. Each question is presented on five-point Likert Scale from strongly disagree (1) to strongly agree (5).

| **Questions** | 1 | 2 | 3 | 4 | 5 |
| --- | --- | --- | --- | --- | --- |
| **Digital Strategy** | | | | | |
| Digitalization is among the top three most important elements of our business strategy. |  |  |  |  |  |
| We investigate the newest trends and future scenarios in digitalization to stay competitive. |  |  |  |  |  |
| Digital projects have a high priority within our business. |  |  |  |  |  |
| We constantly update and refine our digital strategy. |  |  |  |  |  |
| Our competition as well as industry experts perceive us as a leader in digital innovation. |  |  |  |  |  |
| **Digitalization (Overall degree of digitalization)** | | | | | |
| We fully adopt digital artifacts (products or services). |  |  |  |  |  |
| We fully adopt digital platforms that support digital products and services. |  |  |  |  |  |
| We fully adopt digital infrastructures, such as technology tools and systems. |  |  |  |  |  |
| We fully adopt digital business models. |  |  |  |  |  |
| We fully adopt digital management models. |  |  |  |  |  |
| **Digitalization (Adoptation of digital technologies)** | | | | | |
| Big data technology (such as big database, data analysis technology). |  |  |  |  |  |
| AI technology (such as machine learning). |  |  |  |  |  |
| Mobile technology (such as mobile Internet, wireless communications). |  |  |  |  |  |
| Cloud computing technology (such as cloud computing). |  |  |  |  |  |
| IoT technology (such as network distribution technology). |  |  |  |  |  |
| Social technology (such as online commerce, instant messaging). |  |  |  |  |  |
| Platform development technology (such as network platforms). |  |  |  |  |  |
| **Digitalization (Business mode)** | | | | | |
| Your firm’s business of is mainly online. |  |  |  |  |  |
| Your firm’s business of is mainly offline. |  |  |  |  |  |
| **Digitalization (Digital product/services)** | | | | | |
| We exploit all opportunities for digitalization in the market. |  |  |  |  |  |
| We successfully implemented new digital business ideas or business models within the last three years. |  |  |  |  |  |
| The degree of digitalization of our products and services is high compared to our competitors. |  |  |  |  |  |
| We actively integrate customers in the development of digital innovations. |  |  |  |  |  |
| We are able to quickly adapt our digital offerings based on customer feedback. |  |  |  |  |  |
| **Digitalization (Digital services)** | | | | | |
| We implement the most current digital channels (including mobile and social media) in our communication and service processes. |  |  |  |  |  |
| We define and control metrics and goals for our digital channels. |  |  |  |  |  |
| We improve our core processes with the support of digital technologies. |  |  |  |  |  |
| We use the most current digital technology to support standard processes. |  |  |  |  |  |
| We support our decision making by using data analytics |  |  |  |  |  |
| **Environmental Performance** | | | | | |
| Reduction of air emission. |  |  |  |  |  |
| Reduction of hazardous waste/scrap. |  |  |  |  |  |
| Reduction in consumption of gasoline/fuel |  |  |  |  |  |
| Partnership with green organizations and suppliers |  |  |  |  |  |
| Improvement of environmental compliance. |  |  |  |  |  |
| Use of environmental friendly material. |  |  |  |  |  |

Thank you for your participation.
